# Ethnic inequalities in health intervention coverage among Mexican women at the individual and municipality levels

**DOI:** 10.1016/j.eclinm.2021.101228

**Published:** 2021-12-03

**Authors:** Nancy Armenta-Paulino, Fernando C Wehrmeister, Luisa Arroyave, Aluísio J.D. Barros, Cesar G. Victora

**Affiliations:** International Center for Equity in Health, Universidade Federal de Pelotas, Rua Marechal Deodoro, 1160, 3rd floor. 96020-220, Pelotas, Brazil

**Keywords:** ethnic inequalities, Indigenous, women's health, México, SDGs

## Abstract

**Background:**

Using data from Mexico, the country with the largest indigenous population in Latin America, we describe ethnic inequalities in coverage with women's health interventions at individual and municipal levels.

**Methods:**

Cross-sectional study using data from the National Health and Nutrition Survey 2018 and the Mexican Intercensal Survey 2015. We selected five outcomes: modern contraceptive use, content-qualified antenatal care (ANCq), and skilled birth attendant (SBA) for women aged 15–49 years; Pap smear test and mammogram among women aged 25-64 and 40-69 years respectively. Municipalities were classified into three groups by the percentage of indigenous population: <10%, 10% – 39%, and ≥40%. We calculated crude and adjusted coverage ratios (CR) and 95% confidence intervals (CI) using Poisson regression.

**Findings:**

Women living in municipalities with indigenous population ≥40% were poorer, less educated, and more rural. Coverage was lower for indigenous than non-indigenous for modern contraceptive use (CR: 0·73; CI 0·65-0·83), ANCq (CR: 0·72; CI 0·62-0·83), SBA (CR: 0·83; CI 0·77-0·90) and undergoing a mammogram (CR: 0·54; CI 0·41-0·71), but not for Pap smears (CR: 0·94; CI 0·83-1·07). Coverage with the five interventions increased as the municipal proportions of indigenous population decreased, both for indigenous and non-indigenous women. Coverage gaps at municipal level tended to be wider than at individual level.

**Interpretation:**

Both indigenous and non-indigenous women living in municipalities with high proportions of indigenous people were systematically excluded from reproductive and maternal interventions. Our findings suggest that social and health interventions targeted at the individual level should be complemented by structural interventions in municipalities with high proportions of indigenous people, including strengthening health and social services.

**Research in context**

**Evidence before this study**

In Mexico, indigenous people represent 15·1 percent of the total population, and indigenous women are especially vulnerable because they suffer triple discrimination for being women, indigenous and poor. Previous studies have documented lower coverage with maternal interventions, particularly for contraception and skilled birth attendance, as well as for cervical and breast cancer screening. Some studies suggest that ethnic residential concentration affects social interaction structuring as well as access to services and economic resources, but there are no analyses comparing the roles of individual ethnicity and of municipal-level ethnic composition on coverage of women's health services.

**Added value of this study**

Using data from two national surveys, we describe ethnic inequalities in coverage with five women's health interventions, both at individual and municipal levels. The latter are based on a classification of municipalities according to their percentage of indigenous inhabitants. The interventions included modern contraception, antenatal care, birth attendance and screening for cervical and breast cancer. We show that although ethnicity was associated with lower health intervention coverage at individual level, the percentage of indigenous population in the municipality was a more important determinant of coverage, affecting both indigenous and non-indigenous women.

**Implications of all the available evidence**

Women living in municipalities with higher proportions of indigenous people were systematically excluded. Our approach may help countries to analyze and monitor ethnic inequalities according to not only individual but also contextual characteristics. Social and health interventions targeted at the individual level should be complemented by structural interventions in municipalities with high proportions of indigenous people, including strengthening health and social services.

Alt-text: Unlabelled box

## 1 Introduction

The recently published report of the Commission of the Pan American Health Organization on Equity and Health Inequalities in the Americas highlighted the importance of ethnicity as a determinant of health in the Americas[1]. In Latin America, indigenous people are often economically and socially disadvantaged, and have historically been among the poorest in most countries [2,3]. Indigenous communities also present worse health outcomes, lower life expectancy, and limited access to education, healthcare services, and social protection [3].

Indigenous women are especially vulnerable with a triple disadvantage due to ethnicity, gender and being poor [4]. They present higher morbidity and mortality rates than non-indigenous women, because of multiple social determinants including discrimination and limited access to health interventions. In addition, indigenous women are often reluctant to attend health services because of previous negative experiences in dealing with health personnel who do not share their culture and customs [4,5].

The role of place or space in the determination of health status has been an object of epidemiological research for centuries, as part of the triad of persons, places and time [6]. Space or place plays an important role in the health status of populations, as neither the quantity nor the qualit [7]. of health services are uniformly distributed in different geographical areas. Studies have documented that ethnic residential concentration affects both the structuring of social interactions and access to services and economic resources [8,9]. Residential segregation and presence of ethnic enclaves are associated with increased levels of deprivation, which contribute to worse health outcomes [10]. The spatial distribution of ethnic groups produces inequalities in access to services, employment, and life opportunities [9].

In Mexico, indigenous communities are socioeconomically disadvantaged compared to the rest of the population [3,11]. The National Population Council established in 2004 a classification of Mexican municipalities and localities according to their level of concentration of indigenous population [12]. Analyses using this classification of municipalities found that higher indigenous presence was associated with remote location, limited access to healthcare services, and high poverty rates [11,13].

Using the case of Mexico, the country with the largest indigenous population in Latin America, the objective of our analyses was to describe ethnic inequalities in coverage of women's health interventions. We took advantage of the existence of two national surveys in 2015 and 2018 to explore ethnic gaps at the level of individual women, and according to the proportion of indigenous populations at municipal level. Our analyses are the first to attempt to address the roles of individual ethnicity and of municipal-level ethnic composition on coverage of women's health services. We also investigated whether ethnic gaps could be explained by mediating factors such as education, residence, health care affiliation and household wealth.

## 2 Methods

### 2.1 Data sources

This is a cross-sectional study using data from the National Health and Nutrition Survey (ENSANUT) 2018. The ENSANUT used a probabilistic, stratified, two-stage cluster design sample that is representative both at national and state levels and urban/rural strata [14]. We also used data from the Mexican Intercensal Survey 2015 to estimate the proportion of indigenous people by the municipality. The Mexican Intercensal Survey 2015 is a nationwide survey that provides a sample-based snapshot of the population and household composition between the last Mexican census, conducted in 2010, and the next census would be conducted in 2020. It provides information from 2,457 municipalities in the country at the time of the survey [15].

Other data for the municipality characteristics were obtained from the Measurement of municipal poverty 2015 by the National Council for the Evaluation of Social Development Policy (CONEVAL, Consejo Nacional de Evaluación de la Política de Desarrollo Social), and the healthcare facilities dataset (Catálogo CLUES) from Mexican ministry of health [16,17]. This study follows the Strengthening the Reporting of Observational Studies in Epidemiology (STROBE) guidelines.

### 2.2 Coverage indicators

We assessed five indicators of coverage with women's health interventions. For women of reproductive age (15 to 49 years), we analyzed modern contraceptive use, content-qualified antenatal care (ANCq) and skilled birth attendance (SBA). For all adult women we studied cervical screening with the Pap test (25-64 years) and breast cancer screening with mammogram (40-69 years).

Modern contraceptive use was defined as women (married or in union) who were using (or whose partner was using) any modern contraceptive method. The following methods were classified as modern: condoms (male and female), oral contraceptives (pills and the day-after pill), injectables, patches, intrauterine devices, implants (e.g., Norplant), diaphragm, spermicidal agents (foam or jelly), and sterilization (male and female). Other methods, including all calendar methods, were classified as traditional.

Antenatal care was assessed with ANC [18] a content-qualified antenatal care coverage indicator that combines aspects of contact with services with quality of care variables among who delivered a child in the five years before the survey. ANCq is a discrete score ranging from 0 to 10 based on seven variables: first visit in the first trimester of pregnancy (1 point), at least one visit with a skilled provider (2 points), total number of visits (1 point for 1–3 visits, 2 points for 4–7 visits, and 3 points for 8+ visits), blood pressure measured (1 point), blood sample collected (1 point), urine sample collected (1 point), and receiving at least one shot of tetanus toxoid (1 point). The ANCq has been validated, showing significant inverse associations with neonatal mortality [18]. We coded ANCq as a binary variable considering 9 or more ANCq points as an indicator of adequate antenatal care coverage because the national average was 9·1 points.

Skilled birth attendance (SBA) coverage was defined as women who had delivered a child in the five years before the survey who reported that the last delivery was attended by a doctor or nurse as an indicator.

Cervical cancer screening (Pap smear test) coverage, was defined as women aged 25-64 years who reported undergoing screening for cervical cancer in the year before the survey, divided by all women in this age group included in the survey. The Mexican cervical cancer screening guideline recommends that health workers should invite all women in this age range to undergo screening, especially those with risk factors [19]. Women whose first two annual tests are negative should be examined every three years; otherwise, annual exams are recommended. Breast cancer screening (mammography), was defined as women aged 40-69 years who had undergone a mammogram in the past year, divided by all women in this age group included in the survey. This age group is in accordance with the Mexican breast cancer screening guideline [20].

### 2.3 Definition of indigenous status

For the individual level analyses, a woman was identified as indigenous if she reported speaking an indigenous language. Using data from the Mexican Intercensal Survey 2015, we grouped municipalities according to the proportion of inhabitants older than five years who speak an indigenous language into three groups: <10%, 10% – 39%, and ≥40% [12].

### 2.4 Covariates

We analyzed women's characteristics and intervention coverage at the individual and municipal levels. Individual sociodemographic variables included ethnicity, age, marital status (unmarried or married/in union), area of residence (rural or urban), education level, health insurance (affiliation with Mexico's Seguro Popular, social security or private insurance), and household wealth (based on an asset index). The last four variables were treated as mediators (rather than confounders) because according to the social determinants of health framework ethnicity is a distal determinant that affects other sociodemographic variables [1]. Our objective, when adjusting for potential mediators, was to investigate whether these covariates would eliminate the ethnic gaps observed in the unadjusted analyses.

We also described the three groups of municipalities (according to proportion of indigenous population) in terms of the percentage of the population living in poverty, public healthcare facilities (primary to tertiary care) per 10,000 inhabitants, and municipal population.

### 2.5 Data management

Information on the municipal-level proportions of indigenous population from the 2015 Intercensal Survey were added to the individual-level database from ENSANUT 2018, thus allowing comparisons of groups of municipalities as well of individual women.

The ENSANUT collected data from 779 municipalities, but the identification code was unavailable for nine of these due to confidentiality issues. For one municipality, we had no information about the percentage of people who speak an indigenous language from the Mexican intercensal survey because the municipality was created after 2015. Therefore,769 municipalities were analyzed (Supplementary material – Table S1).

### 2.6 Statistical analysis

Coverage ratios (CR) were calculated for indigenous compared with non-indigenous women within the three groups of municipalities using Poisson regression for outcomes coded as binary variables [21,22]. This approach has the advantage of providing results as prevalence ratios, which are more intuitive and easily interpreted than other association measures such as odds ratios from logistic regression [21]. The robust variance option for Poisson regression ensures the assumptions behind the regression model are not violated. We used crude models to assess how much coverage varied by individual ethnicity and adjusted models to investigate whether ethnic gaps in coverage could be explained by differences in terms of sociodemographic covariates described above. We also tested for interaction between individual-level ethnicity and municipal proportions of indigenous populations in the Poisson regression. All analyses considered the survey design and were performed in Stata (StataCorp. 2019. Stata Statistical Software: Release 16. College Station, TX: StataCorp LLC.).

### 2.7 Ethical approval

All analyses relied on publicly available anonymized databases. Ethical approval was obtained by the national institutions responsible for each survey. Instituto Nacional de Salúd Pública (INSP) for the ENSANUT 2018 and Instituto Nacional de Estadística, Geografía e Informática (INEGI) for the Mexican Intercensal Survey 2015.

### 2.8 Role of the funding source

The funders of the study had no role in study design, data collection, data analysis, data interpretation, or writing of the report.

## 3 Results

We classified the 769 municipalities surveyed according to their indigenous population percentage: 10% had ≥ 40%, 9% among 10%-39%, and 81% <10%. The numbers of municipalities and women in each group are shown in the Supplementary materials (Table S1). Mexico's indigenous population is highly concentrated in the southern and south-central regions (Supplementary material – Figure S1).

Table 1 shows the distributions of women's individual characteristics according to the municipal proportion of indigenous population. In general, women living in municipalities with ≥40% indigenous population were highly concentrated in the poorest wealth quintiles (69·3%), mainly residing in rural areas (58·7%) and having lower educational levels (14·8% without education and 36·2% just primary). Due to targeting of health insurance at poorer population groups (Mexico's Seguro Popular), coverage was higher in municipalities with greater indigenous presence. At the bottom of Table 1, we compare selected municipal characteristics among the three groups. The population living in poverty was the highest (82·5%) among municipalities with stronger indigenous presence, but health facilities per population were more common in such municipalities.

**Table 1:** Sociodemographic characteristics of women aged 15-69 years according to municipal percentage of indigenous population and municipal-level demographic and health care characteristics

Characteristics	Proportion of indigenous population (95% IC)		
	<10%	10-39%	≥40%
Municipalities	622	72	75
**Individual-level**			
Ethnicity			
Indigenous	1·7 (1·4;2·0)	20·1 (14·8;26·7)	68·5 (61·7;74·5)
Non-indigenous	98·3 (98·0;98·6)	79·9 (73·3;85·2)	31·5 (25·5;38·3)
Age (mean)	38·6 (38·3;38·9)	37·7 (36·8;38·6)	37·1 (35·9;38·3)
Area of residence			
Rural	18·0 (17·1;18·9)	30·9 (24·2;38·5)	58·7 (49·4;67·4)
Urban	82·0 (81·1;82·9)	69·1 (61·5;75·8)	41·3 (32·6;50·6)
Education level			
No education	3·3 (3·0;3·7)	5·7 (4·3;7·7)	14·8 (11·9;18·4)
Primary	20·3 (19·5;21·2)	22·9 (20·2;25·8)	36·2 (32·6;39·9)
Secondary	30·5 (29·5;31·4)	30·3 (27·5;33·3)	27·6 (24·4;31·0)
Middle higher	25·4 (24·5;26·3)	24·4 (22·0;26·9)	15·5 (12·6;18·9)
Higher	20·5 (19·6;21·3)	16·7 (14·3;19·4)	5·9 (4·0;8·7)
Seguro Popular insurance			
No	17·7 (16·8;18·6)	16·9 (14·5;19·7)	10·2 (8·1;12·8)
Yes	82·3 (81·4;83·2)	83·1 (80·3;85·5)	89·8 (87·2;91·9)
Marital status			
Unmarried	43·0 (41·9;44·0)	39·9 (37·0;42·9)	37·1 (33·8;40·6)
Married or union	57·0 (56·0;58·1)	60·1 (57·1;63·0)	62·9 (59·4;66·2)
Wealth quintiles (asset index)			
Q1 (poorest)	13·5 (12·7;14·3)	26·7 (22·0;32·0)	69·3 (63·2;74·8)
Q2	19·2 (18·4;20·1)	23·3 (20·2;26·7)	18·2 (15·4;21·4)
Q3	21·3 (20·4;22·2)	19·3 (16·8;22·1)	7·2 (5·4;9·6)
Q4	22·2 (21·3;23·2)	19·7 (16·4;23·4)	4·1 (2·5;6·7)
Q5 (wealthiest)	23·8 (22·8;24·8)	11·1 (9·1;13·4)	1·2 (0·5;2·4)
**Municipal-level**			
Percentage of population living in poverty (mean)	40·7 (40·2;14·3)	51·0 (48·0;20·5)	82·5 (80·6;42·5)
Health care facilities per 10,000 inhabitants (mean)	14·3 (13·7;14·8)	20·5 (17·6;23·5)	42·5 (39·0;46·0)
Municipal population (mean)	496,727 (481,408;512,046)	273,086 (240,045;306,126)	53,126 (44,740;61,511)

**Figure 1:**
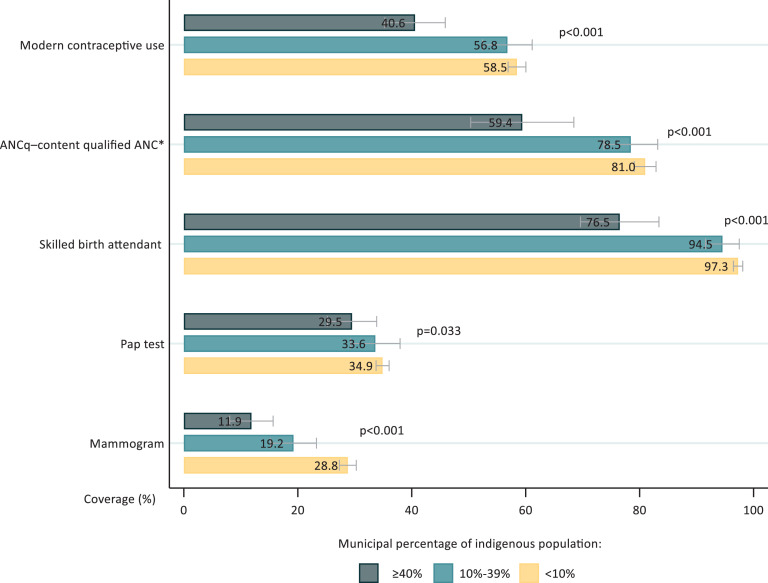
Intervention coverage for all resident women, according to municipal percentage of indigenous population

Note: *Coded as a binary variable: ANCq score ≥ 9 points.

**Figure 2:**
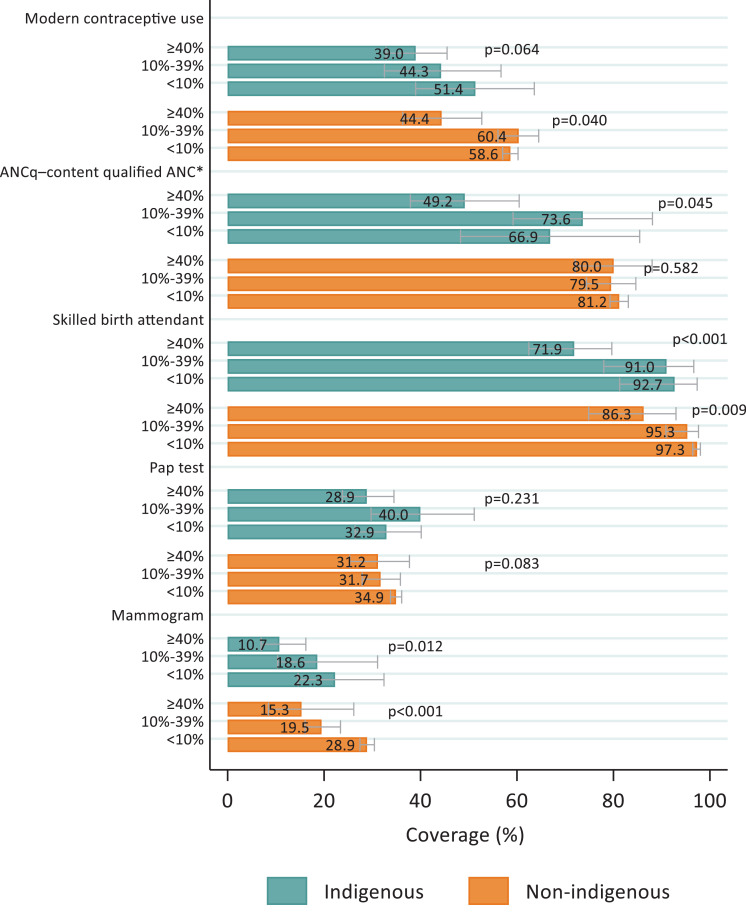
Intervention coverage for indigenous and non-indigenous women, according to municipal percentage of indigenous population

Note: *Coded as a binary variable: ANCq score ≥ 9 points.

Figure 1, Figure 2, and Tables S2 and S3 (Supplementary materials) show the municipal level analyses. Coverage with the five interventions increased as the municipal proportions of indigenous population decreased. Tests for linear trends were significant for all interventions (Figure 1). Differences among the extreme categories were close to 20 percent points for modern contraceptive use, ANCq, SBA and mammograms.

We also analyzed how coverage among indigenous and non-indigenous women varied according to the municipal proportions of indigenous populations. Figure 2 and Table S3 show that the municipal coverage gradient affected both indigenous and non-indigenous women living in these municipalities, particularly for mammograms and SBA. The confidence intervals for both groups of women overlapped in every case, thus suggesting that the proportion of indigenous people in the municipality is more important than the individual's ethnicity.

We also calculated crude and adjusted coverage ratios in indigenous women compared to non-indigenous women (Table 2). In all municipalities combined, coverage was significantly lower for indigenous than non-indigenous women for modern contraceptive use (CR: 0·73; CI 0·65-0·83), ANCq (CR: 0·72, CI 0·62-0·83), SBA (CR: 0·83; CI 0·77-0·90) and undergoing a mammogram (CR: 0·54; CI 0·41-0·71). The corresponding gaps were 15·1, 22·9, 17·6, and 12·6 percent points. Although coverage tended to be slightly lower for indigenous than for non-indigenous women regarding Pap smears, confidence intervals overlapped with a gap of only 2·4 percent points.

**Table 2:** Individual-level crude and adjusted coverage ratios for health interventions in indigenous compared to non-indigenous women (reference group) within each group of municipalities (CI 95%)

Groups of municipalities according to proportion of indigenous population	Maternal health care							Cervical and breast cancer screening			
	Modern contraceptive use		ANC quality score^1^		Skilled birth attendant			Pap smear test		Mammogram	
	Crude	Adjusted^2^	Crude	Adjusted^2^	Crude	Adjusted^2^		Crude	Adjusted^2^	Crude	Adjusted^2^
All municipalities	0·73 (0·65;0·83)	0·84 (0·74;0·95)	0·72 (0·62;0·83)	0·81 (0·71;0·93)	0·83 (0·77;0·90)	0·88 (0·82;0·94)		0·94 (0·83;1·07)	1·03 (0·90;1·18)	0·54 (0·41;0·71)	0·76 (0·58;1·00)
<10%	0·88 (0·68;1·12)	0·95 (0·75;1·22)	0·82 (0·64;1·06)	0·88 (0·69;1·12)	0·95 (0·88;1·03)	0·97 (0·91;1·03)		0·94 (0·76;1·16)	1·04 (0·84;1·28)	0·77 (0·51;1·15)	0·97 (0·65;1·44)
10-39%	0·73 (0·54;0·99)	0·78 (0·58;1·04)	0·93 (0·74;1·15)	0·93 (0·75;1·16)	0·95 (0·85;1·07)	0·98 (0·87;1·10)		1·26 (0·97;1·63)	1·23 (0·95;1·59)	0·95 (0·54;1·68)	1·12 (0·71;1·76)
≥40%	0·88 (0·69;1·12)	1·00 (0·77;1·28)	0·61 (0·48;0·79)	0·80 (0·63;1·01)	0·83 (0·71;0·98)	0·95 (0·79;1·15)		0·93 (0·71;1·21)	0·87 (0·67;1·13)	0·70 (0·32;1·53)	0·96 (0·45;2·06)
Test for interaction between individual ethnicity and municipal proportions of indigenous population (p-value)	0·30	0·70	0·14	0·34	0·21	0·51		0·59	0·75	0·20	0·88

Note: 1) Coded as a binary variable: ANCq score ≥ 9 points; 2) Adjusted all for the following individual-level variables: age, area of residence (rural or urban), education level, health insurance (affiliation with Mexico's Seguro Popular, social security or private insurance), marital status (unmarried or married/in union), and household wealth (based on an asset index).

After adjustment for individual sociodemographic characteristics, there was marked attenuation of coverage ratios for all interventions (Table 2), although coverage tended to remain lower for indigenous women. Table 2 also shows individual level coverage ratios stratified by the municipal proportions of indigenous population, but there was no evidence that these ratios varied among the three groups of municipalities. Tests for interactions among individual-level ethnicity and groups of municipalities were not significant, suggesting that the coverage ratios are similar in all groups.

## 4 Discussion

Mexico's health system is segmented across diverse public and private payers and providers. The Federal Ministry of Health and state governments have established provider networks that share responsibility for public health care programs for the entire population as well as social assistance for the uninsured poor. However, these health services are affected by unequal access and quality limitations [23]. The private sector also plays an important role. Even very poor Mexican households have, to varying degrees, geographical access to general physicians who typically provide their low-cost services in consulting rooms adjacent to pharmacies in municipalities that range from small towns to large urban areas [23]. Since the end of 2018, the Mexican Health System and the Mexican Social Policy have been undergoing a major transformation. The government eliminated Seguro Popular in January 2020 and centralized the health system, and ended the *Progresa-Oportunidades-Prospera*, a conditional cash transfer program, which had operated with great continuity for more than twenty years [24,25]. Although these programs did not focus on indigenous population groups, they have had a positive effect on education and health indicators [26,27]. Our results should be interpreted in light of this complex combination of health services.

Our analyses show that ethnic gaps in coverage with selected women's health interventions are present not only at individual level but also – and more importantly – at municipal level, as all women living in municipalities with higher proportions of indigenous people tend to be systematically excluded from receiving key interventions.

Even the non-indigenous women residing in municipalities with a higher proportion of indigenous people had lower coverages for SBA and mammogram than those living in municipalities with <10% of the indigenous population. The corresponding gaps were 11 and 13·6 percent points, respectively. The gap for SBA was even higher than the difference among indigenous women (11·6).

The conceptual model behind our analyses proposed that residential location, reflecting ethnic concentration at municipal level, represents a distal or structural determinant, whereas sociodemographic factors at individual or municipal levels constitute proximate determinants [1,28]. According to the social determinants of health framework [29]. the effects of a distal determinant such as ethnicity can be assessed in cross-sectional designs, given that ethnic group affiliation is defined at conception, whereas health coverage was assessed among adult women. Indeed, the bulk of the equity literature, including determinants such as gender, wealth and ethnicity, is based on cross-sectional analyses [1,29].

Inequalities experienced by indigenous women and communities are intimately associated with prevailing socioeconomic conditions [30]. Our results confirm that municipalities with higher proportions of indigenous people tend to be poorer and less well served by health services than other municipalities [11,^12^,31]. At individual level, indigenous women are poorer, less educated, and have less access to services than non-indigenous women, also have a higher proportion of people living in poverty. For the indigenous people, such structural factors increase their vulnerability to preventable and treatable health conditions [32]. The importance of structural factors is reinforced by the lower coverages observed for some health interventions in non-indigenous women who live in communities with higher proportions of indigenous people.

Because ethnicity is a distal determinant of health and its effects are mediated by poverty, education and place of residence, among other factors, the full effects of ethnic group affiliation are observed in the crude analyses. Adjusted effects solely answer the question of whether the ethnic gaps observed in the crude analyses are explained by the measured mediating factors. [1,28].

Institutional discrimination may affect health by generating ethnic differences in residential environments, socioeconomic position, access to goods and services, and determining access to medical care. Residential segregation constitutes one of the most relevant mechanisms of institutional discrimination, leading to reduced opportunities for education, employment, recreation and exposure to health-promoting environments [30].

Ethnic group segregation is also associated with the retention of cultural identity. Substantial proportions of members of a given ethnic group opt to remain in residential enclaves in order to preserve aspects of their cultural identity [33]. including health care practices for women [34]. There are many examples of limited success in delivering health interventions to indigenous people due to lack of awareness or acceptance of indigenous cultural behaviors, among which are language barriers and respecting the need for families to be present during clinic visits, or for female patients to have female clinical staff in attendance [35]. As a consequence, such cultural barriers result in indigenous women being vulnerable to receiving the substandard quality of care, being subjected to long delays and experiencing shame, humiliation, exclusion, and other forms of human rights violations [36]. Many predominantly indigenous communities in Mexico do have health clinics, albeit – as our results show – at a lower ratio to their population than other municipalities. Yet, such clinics are often occupied by inadequately trained medical students or junior health staff who do not speak the local indigenous languages and can be arrogant towards the women [37].

Our findings show that ethnic inequalities in coverage vary by intervention. The main gaps, both at individual and municipal levels, were observed for modern contraceptive use, content-qualified antenatal care, skilled birth attendance and mammography.

Results for contraception are consistent with findings from earlier analyses that compared indigenous and non-indigenous in Latin American countries [38,39]. Indigenous women had 27% lower coverage than non-indigenous women (a gap of 15·1 percent points) compared to a difference of 17·9 percent points among municipalities with <10% and ≥40% indigenous population. Low coverage in indigenous women has been attributed to poor supply of modern methods, low decision-making ability of women, taboos relating to reproductive health and lack of knowledge regarding modern contraceptives [4,^36^,40]. Adjustment for potential mediating variables attenuated, but did not eradicate, the effects at individual and municipal level.

In terms of antenatal care quality, we observed a gap of 21·6 percent points in ANCq coverage among women living in municipalities with <10% and ≥40% indigenous population (Supplementary material – Table S2). Higher coverages (considering 9 or more ANCq points) indicate that women had both adequate levels of contact with health services and received most if not all recommended interventions during pregnancy. The gaps are important because higher ANCq scores are associated with lower neonatal mortality in the offspring [18]. Also, higher ANCq scores have been reported for women living in urban areas, with secondary or more level of education, belonging to wealthier families and with higher empowerment, showing large inequalities across socioeconomic groups between and within countries [41].

SBA coverage showed a significant gap of 20·8 percent points among municipalities with <10% and ≥40% indigenous population, whereas at individual level the coverage ratio was 0·83 for indigenous women (a gap of 17·6 percent points). These differences practically disappear after adjustment, indicating that most of the observed gap was explained - or mediated - by other structural factors [28]. Indigenous or predominantly indigenous municipalities are markedly disadvantaged in terms of essential health services as well as of socioeconomic characteristics as shown by our data and by earlier studies [13].

The women's cancer screening indicators showed markedly lower coverage than indicators of contraception, antenatal and delivery care. For Pap smears, coverage was 32·3% in indigenous and 34·7% in non-indigenous women with a crude non-significant coverage ratio of 0·94. The corresponding gap among municipalities with <10% and ≥40% indigenous population was of 5·4 percent points. The low coverage levels should be interpreted with caution as the indicator refers to exams in the 12 months preceding the survey. Mexican screening guidelines recommend that women with two consecutive negative Pap tests should only be screened again after three years [19]. so that annual coverage levels likely represent an underestimate. However, there is evidence from other Mexican studies that indigenous women often fail to be screened for cervical cancer [42].

For mammography, the coverage ratio was 0·54 and highly significant, with a gap of 12·6 percent points. The corresponding gaps among municipalities with <10% and ≥40% indigenous population was equal to 16·9 percent points. Although Mexican guidelines recommend a mammography every two years for women aged 40-69 years, our coverage results based on tests during the 12 months before the survey could be underestimated, but even if coverage levels are multiplied by two these are evidently too low. Our results are consistent with low coverage levels identified in previous studies [42,43].

Despite the above-noted limitations with measuring coverage of cancer screening indicators, it is evident that breast and cervical screening present lower coverage than maternal health interventions. The difference is likely due to poor health infrastructure and limited human resources for screening programs, mainly in rural areas [42].

The effects of individual ethnicity and municipal level ethnic distribution were investigated using a multilevel regression model with women as the level 1 and municipality as level 2. These analyses were not helpful because - as expected – the vast majority of indigenous women live in municipalities with ≥40% of indigenous population, and thus simultaneous adjustment for individual and municipal level ethnicity led to major attenuation of the effects observed in both levels due to marked collinearity.

Our analyses have limitations that should be noted. The survey sampling frames were not designed specifically with ethnicity in mind, so it was only possible to consider the indigenous population percentage at the municipality level because the identification of localities was not available in the survey. Some municipalities are made up by several smaller localities which may be heterogeneous in terms of their proportions of indigenous people. Other limitations include the fact that coverage was measured through recall by women, which may lead to under or overreporting; however, this would only affect our analyses if there was differential recall among indigenous and non-indigenous women. Although the ANCq definition and Mexican guidelines for antenatal care consist of at least two shots of tetanus toxoid during pregnancy [18,44]. we estimated the ANCq score for at least one shot because that was the information was recorded in the survey. Still regarding limitations of the indicators, it is unlikely that this difference would explain the observed gaps. Our results at municipal level might have been affected by the ecological fallacy, but it is reassuring that the individual-level analyses showed similar ethnic gaps.

We found relevant inequalities related to the indigenous population presence that need to be addressed. Both indigenous and non-indigenous women living in municipalities with higher proportions of indigenous people were systematically excluded. Our findings suggest that social and health interventions targeted at individual level – such as health insurance and social benefits – should be complemented by structural interventions in municipalities with high proportions of indigenous people, including strengthening health and social services. Considering the commitment of the Sustainable Development Goals towards leaving no one behind and responding to the call to produce data disaggregated by ethnicity in SDG 17.18, [45]. our approach may help countries to analyze and monitor ethnic inequalities according not only individual but also contextual characteristics.

## Data sharing statement

All data used are available publicly on the ENSANUT (https://ensanut.insp.mx/encuestas/ensanut2018/descargas.php), and National Institute of Statistics and Geography (INEGI) websites (https://www.inegi.org.mx/programas/intercensal/2015/).

## Contributions

NAP and CGV conceptualized the study, interpreted the results, and wrote the manuscript. NAP performed the analyses. LA assisted in the analysis, interpreting the results, and writing the manuscript. AJDB critically reviewed the analyses and contributed to writing the manuscript. FCW critically reviewed and edited the manuscript. All authors had access to all data used in the study. NAP and LA accessed and verified the underlying study data. All authors had final responsibility for the decision to submit for publication.

## Funding

This paper was made possible with funds from Bill & Melinda Gates Foundation (Grant Number: INV-010051 / OPP1199234), Wellcome Trust (Grant 101815/Z/13/Z), and Associação Brasileira de Saúde Coletiva (ABRASCO).

## Declaration of Competing Interest

We declare no competing interests.

## Appendix Supplementary materials

Image, application 1

Image, application 2
