# Supplementary material for: Ethnic inequalities in health intervention coverage among Mexican women at the individual and municipality levels
Source: eClinicalMedicine. 2021 Dec 3;43:101228. doi: 10.1016/j.eclinm.2021.101228 (PMC8649218; doi:10.1016/j.eclinm.2021.101228)
Supplement: Supplementary file 1 [file mmc1.docx]

**Supplementary material**

Table S1. Municipalities analyzed

Figure S1. Percentage of indigenous people in Mexico Municipalities, 2015

Table S2. Women's health interventions coverage according to the indigenous population percentage in the municipality (CI 95%)

Table S3. Women's health interventions coverage indigenous and non-indigenous according to the indigenous population percentage in the municipality (CI 95%)
