# Supplementary material for: Ethnic inequalities in health intervention coverage among Mexican women at the individual and municipality levels
Source: eClinicalMedicine. 2021 Dec 3;43:101228. doi: 10.1016/j.eclinm.2021.101228 (PMC8649218; doi:10.1016/j.eclinm.2021.101228)
Supplement: Supplementary file 2 [file mmc2.docx]

**Supplementary material**

# **Table S1. Municipalities analyzed**

| Women living in municipalities by the indigenous population percentage | Municipalities ENSANUT 2018^a^ | Women aged 15-49 years who had a birth in the last 5 years | | Women aged  25-64 years | | Women aged  40-69 years | |
| --- | --- | --- | --- | --- | --- | --- | --- |
|  |  | Municipalities | N | Municipalities | N | Municipalities | N |
| <10% | 622 | 568 | 3980 | 620 | 15,264 | 616 | 9,650 |
| 10-39% | 72 | 64 | 365 | 72 | 1,380 | 72 | 843 |
| ≥40% | 75 | 69 | 330 | 75 | 896 | 75 | 505 |
| Total | 769 | 701 | 4,675 | 767 | 17,540 | 763 | 10,998 |

Notes: a) The identification code was not available for nine municipalities due to confidentiality issues, and for one, we have no information about the % of people who speak an indigenous language.

# **Figure S1. Percentage of indigenous people in Mexico Municipalities, 2015**


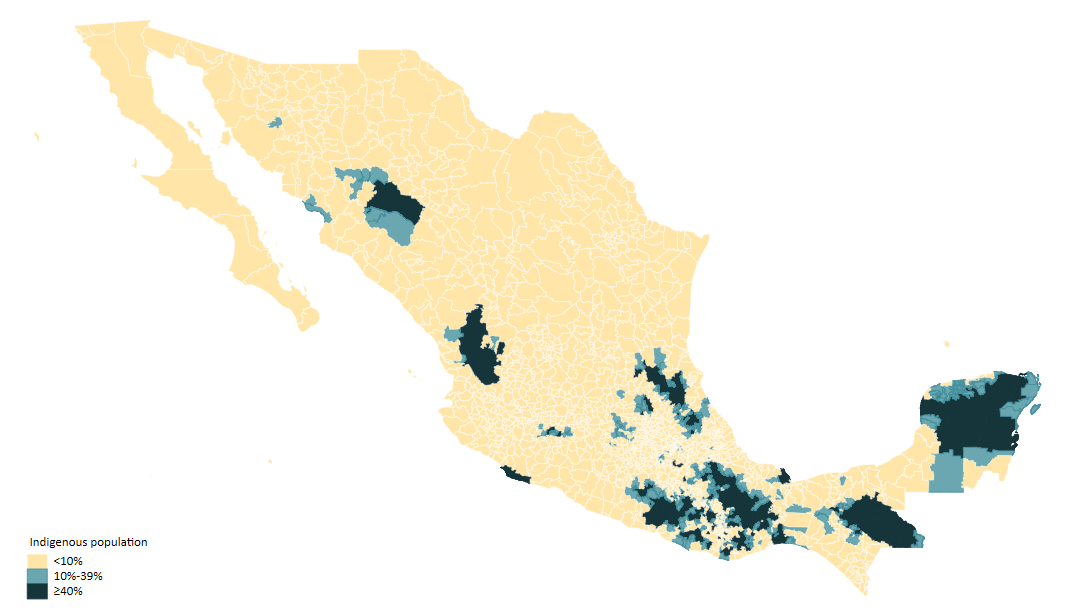


# **Table S2. Women's health interventions coverage according to the indigenous population percentage in the municipality (CI 95%)**

| Women living in municipalities by the indigenous population percentage | Maternal health interventions | | | |  | Cervical and Breast Cancer Screening | |
| --- | --- | --- | --- | --- | --- | --- | --- |
|  | Modern contraceptive use | ANCq^1^ |  | Skilled birth attendant |  | Pap test | Mammogram |
| <10% | 58·5 (56·9;60·0) | 81·0 (79·1;82·9) |  | 97·3 (96·3;98·0) |  | 34·9 (33·7;36·0) | 28·8 (27·3;30·3) |
| 10-39% | 56·8 (52·4;61·1) | 78·5 (73·7;83·2) |  | 94·5 (90·7;96·8) |  | 33·6 (29·4;38·1) | 19·2 (15·5;23·6) |
| ≥40% | 40·6 (35·3;45·9) | 59·4 (50·4;68·5) |  | 76·5 (69·6;83·4) |  | 29·5 (25·2;33·8) | 11·9  (8·1;15·7) |

Note: 1) Coded as a binary variable: ANCq score≥ 9 points

# **Table S3. Women's health interventions coverage indigenous and non-indigenous according to the indigenous population percentage in the municipality (CI 95%)**

| Women living in municipalities by the indigenous population percentage | Maternal health interventions | | | | | | Cervical and Breast Cancer Screening | | | |
| --- | --- | --- | --- | --- | --- | --- | --- | --- | --- | --- |
|  | Modern contraceptive use | | ANCq^1^ | | Skilled birth attendant | | Pap test | | Mammogram | |
|  | Indigenous | Non-indigenous | Indigenous | Non-indigenous | Indigenous | Non-indigenous | Indigenous | Non-indigenous | Indigenous | Non-indigenous |
| All municipalities | 43·4  (38·1;48·9) | 58·5  (57·0;60·0) | 58·2 (49·8;66·5) | 81·1 (79·3;82·8) | 79·4 (72·4;85·0) | 97·0  (96·2;97·7) | 32·3  (28·1; 36·7) | 34·7  (33·6;35·8) | 15·8  (12·0;20·4) | 28·4  (26·9·4;29·8) |
| <10% | 51·4  (39·0;63·6) | 58·6  (57;60·2) | 66·9 (48·3;85·5) | 81·2 (79·3;83·1) | 92·7  (81·3;97·4) | 97·4  (96·5;98) | 32·9  (26·5;40·1) | 34·9  (33·8;36·1) | 22·3  (14·6;32·4) | 28·9  (27·4;30·4) |
| 10% – 39% | 44·3  (32·5;56·7) | 60·4  (56·1;64·5) | 73·6 (59·2;88·1) | 79·5 (74·4;84·6) | 91·0  (78;96·7) | 95·3  (90·9;97·6) | 40·0  (29·7;51·1) | 31·7  (27·9;35·8) | 18·6  (10·4;31·1) | 19·5  (16·1;23·4) |
| ≥40% | 39·0  (32·9;45·5) | 44·4  (36·3;52·7) | 49·2 (37·9;60·5) | 80·0 (72·1;88·0) | 71·9  (62·5;79·7) | 86·3  (74·9;93) | 28·9  (23·8;34·5) | 31·2  (25·3;37·7) | 10·7  (6·9;16·2) | 15·3  (8·5;26·2) |

Note: 1) Coded as a binary variable: ANCq score≥ 9 points
